# m6A RNA Methylation Regulators Participate in the Malignant Progression and Have Clinical Prognostic Value in Lung Adenocarcinoma

**DOI:** 10.3389/fgene.2020.00994

**Published:** 2020-08-25

**Authors:** Fangwei Li, Hong Wang, Huirong Huang, Li Zhang, Dan Wang, Yixin Wan

**Affiliations:** Department of Respiratory Medicine, Lanzhou University Second Hospital, Lanzhou, China

**Keywords:** m6A, lung adenocarcinoma, methylation, demethylation, prognostic signature

## Abstract

Abnormal methylation of N6 adenosine (m6A) in RNA plays a crucial role in the pathogenesis of many types of tumors. However, little is known about m6A RNA methylation in lung adenocarcinoma. This study aimed to identify the value of m6A RNA methylation regulators in the malignant progression and clinical prognosis of lung adenocarcinoma. The RNA-seq transcriptome data and corresponding clinical information of lung adenocarcinoma were downloaded from The Cancer Genome Atlas (TCGA) and Genotype-Tissue Expression (GTEx) database. Then the identification of differentially expressed m6A RNA methylation regulators between cancer samples and normal control samples, different subgroups by consensus expression of these regulators and the prognostic signature were achieved using R software with multiple corresponding packages. The results showed that the expression levels of HNRNPC, YTHDF1, KIAA1429, RBM15, YTHDF2, and METTL3 in cancer group were significantly up-regulated (*P* < 0.05), while expression levels of FTO, ZC3H13, METTL14, YTHDC1 and WTAP in cancer group were significantly down-regulated (*P* < 0.05) compared with control group. Two subgroups identified by consensus expression of these regulators were closely related to the clinicopathological features, clinical outcomes and malignancy of lung adenocarcinoma. In addition, a 3-gene risk signature including KIAA1429, RBM15, and HNRNPC was constructed and the lung adenocarcinoma patients in TCGA database were divided into high-risk group and low-risk group based on the median risk score. In conclusion, the prognostic signature-based risk score calculated according to the expression levels of KIAA1429, RBM15, and HNRNPC, was not only strongly associated with clinical outcomes and clinicopathological features, but also an independent prognostic factor in lung adenocarcinoma.

## Introduction

Lung adenocarcinoma is an aggressive disease which derives from small airway epithelial or type II alveolar cells, accounting for approximately 40% of all lung cancer cases as the most common type (Noguchi et al., 1995; Zappa and Mousa, 2016). The evolution of lung adenocarcinoma is a multistep process which involves genetic, epigenetic and environmental factor interactions, resulting in the dysregulation of key oncogenes as well as tumor suppressor genes and subsequent activation of cancer-related signaling pathways (Ansari et al., 2016). Multiple molecular aberrations driving lung adenocarcinoma development have been discovered in the past decade, among which EGFR and ALK gene mutations are the most important ones (Devarakonda et al., 2015). However, alternative therapeutic strategies for treating lung adenocarcinoma underscore their importance due to the absence of target gene mutation in a significant proportion of lung adenocarcinoma and the resistance of new targeted drugs (Minguet et al., 2016). Among these strategies, epigenetics, especially RNA methylation has drawn wide attention (Chao et al., 2020).

Methylation of N6 adenosine (m6A) in RNA is a methylation modification that occurs on the sixth N atom of RNA adenine, which is highly conserved and extensively occurs in most eukaryotic species, playing a vital role in post-transcriptional regulation (Wang and Zhao, 2016; Duan et al., 2019). m6A RNA methylation is a dynamic reversible process, which is regulated by methyltransferases (writers), demethylases (erasers) and binding proteins (readers) (Yang et al., 2018). “Writers,” including METTL3, METTL14, KIAA1429, WTAP, RBM15, and ZC3H13, mediate the process of methylation modification of RNA; “erasers,” such as FTO and ALKBH5, remove the RNA methylation modification signal; “readers,” consist of YTHDC1, YTHDC2, YTHDF1, YTHDF2, and HNRNPC, recognize those m6A-modified RNAs to regulate the expression of various genes (Tong et al., 2018).

m6A RNA methylation is involved in diverse biological processes, such as differentiation and pluripotency of stem cells, embryogenesis, DNA damage response as well as the transcription, alternative splicing, nuclear export, translation and degradation of mRNAs (Batista et al., 2014; Xiang et al., 2017; Zhao et al., 2017; Sun et al., 2019). More and more studies have shown that abnormal m6A RNA methylation plays a crucial role in the pathogenesis of many human diseases including cancers (Liu et al., 2018; De Jesus et al., 2019; Devaux and Nossent, 2019; Gokhale et al., 2019; Liu X. et al., 2019). However, little is known about m6A RNA methylation in lung adenocarcinoma. In this study, we systematically examined the expression of 13 widely reported m6A RNA methylation regulators in 535 lung adenocarcinoma tissues from TCGA database, analyzed the relationship between these regulators and clinicopathological features, and built a 3-gene risk signature, which was indicated an independent prognostic factor for lung adenocarcinoma.

## Materials and Methods

### Data Acquisition

The RNA-seq transcriptome data and corresponding clinical information of lung adenocarcinoma cohort were downloaded from TCGA data portal: The National Cancer Institute Genomic Data Commons [21]. In addition, more RNA-seq transcriptome data of normal lung samples were obtained from Genotype-Tissue Expression (GTEx) database using UCSC Xena. RNA-seq by expectation maximization (RSEM) approach was used to normalize the transcriptome data. Finally, a total of 535 lung adenocarcinoma tissues and 347 normal lung tissues in TCGA and GTEx database were included for subsequent analysis.

### Selection and Differential Expression Analysis of m6A RNA Methylation Regulators

A total of 13 m6A RNA methylation regulators were obtained from published literatures, including METTL3, YTHDF1, KIAA1429, YTHDC2, ALKBH5, YTHDF2, YTHDC1, ZC3H13, METTL14, FTO, WTAP, RBM15, and HNRNPC.

We extracted the expression matrix of these 13 genes as well as clinical information of 535 lung adenocarcinoma samples and 347 normal lung samples in TCGA and GTEx database. Then the differentially expressed m6A RNA methylation regulators between the cancer group and the control group were identified through R software (version 3.6.1) with limma package. *P*-value < 0.05 and | log_2_ (fold change)| > 1 were considered to be significantly different. Subsequently, a heat map and a violin plot were used to display the differential expression of m6A RNA methylation regulators between the two groups.

### PPI Network Construction and Correlation Analysis

The protein-protein interactions (PPI) among different m6A RNA methylation regulators were analyzed using String database [22]. The association among these genes was revealed by Pearson correlation analysis, which was performed in R software with corrplot package.

### Consensus Clustering of m6A RNA Methylation Regulators

To investigate the function of m6A RNA methylation regulators in lung adenocarcinoma, the lung adenocarcinoma cohort in TCGA database was clustered into different subgroups by consensus expression of these regulators. This grouping result was achieved with ConsensusClusterPlus package, verified using PCA with limma package and visualized with ggplot2 package in R software.

### Relationship Exploration Between Consensus Clustering and the Clinicopathological Features, Clinical Outcomes as Well as Malignancy of Lung Adenocarcinoma

To explore the relationship between consensus clustering and the clinicopathological features, clinical outcomes as well as malignancy of lung adenocarcinoma, chi-square test was firstly used to compare the distribution of age, gender, survival state and stage among different clusters, and the results were presented using pheatmap package in R software. Next, the OS difference among different clusters was calculated by the Kaplan–Meier method and visualized using survival package in R software. Finally, GO and KEGG pathway enrichment analysis of genes that were differently expressed among different clusters were performed using clusterProfiler package and org.Hs.eg.db package, respectively, in R software.

### Identification of Prognostic Signature

The correlation between each m6A RNA methylation regulator and OS of lung adenocarcinoma patients in TCGA database was evaluated using univariate Cox analysis in R software. Regulators with a value of HR larger than 1 were considered as risky genes, while those with a value of HR less than 1 were regarded as protective genes. According to the result, 3 genes were identified significantly related to OS with the *P*-value < 0.05, which were further chosen for the construction of prognostic signature. Then the coefficients of 3 genes were determined by LASSO algorithm and the risk score of each patient for prognostic signature was calculated using the following formula: Risk score = coefficient 1 ^∗^ value 1 + coefficient 2 ^∗^ value 2 + coefficient 3 ^∗^ value 3, where value was the relative expression level of each selected gene. Finally, the lung adenocarcinoma patients in TCGA database were divided into high-risk group and low-risk group based on the median risk score.

### Evaluation of the Relationship Between Prognostic Signature-Based Risk Score and Clinical Outcomes as Well as Clinicopathological Features of Lung Adenocarcinoma

To evaluate the relationship of prognostic signature-based risk score and clinical outcomes of lung adenocarcinoma, the difference of OS between high-risk group and low-risk group was calculated by Kaplan–Meier method. Then chi-square test was performed to compare the distribution of clinicopathological parameters between the two groups and a heatmap was used to visualize the results. In addition, univariate and multivariate Cox regression analysis were used to identify whether age, gender, stage or risk score was an independent prognostic factor for lung adenocarcinoma in TCGA database.

## Results

### The Differentially Expressed m6A RNA Methylation Regulators Between Lung Adenocarcinoma Samples and Normal Control Samples

**FIGURE 1:**
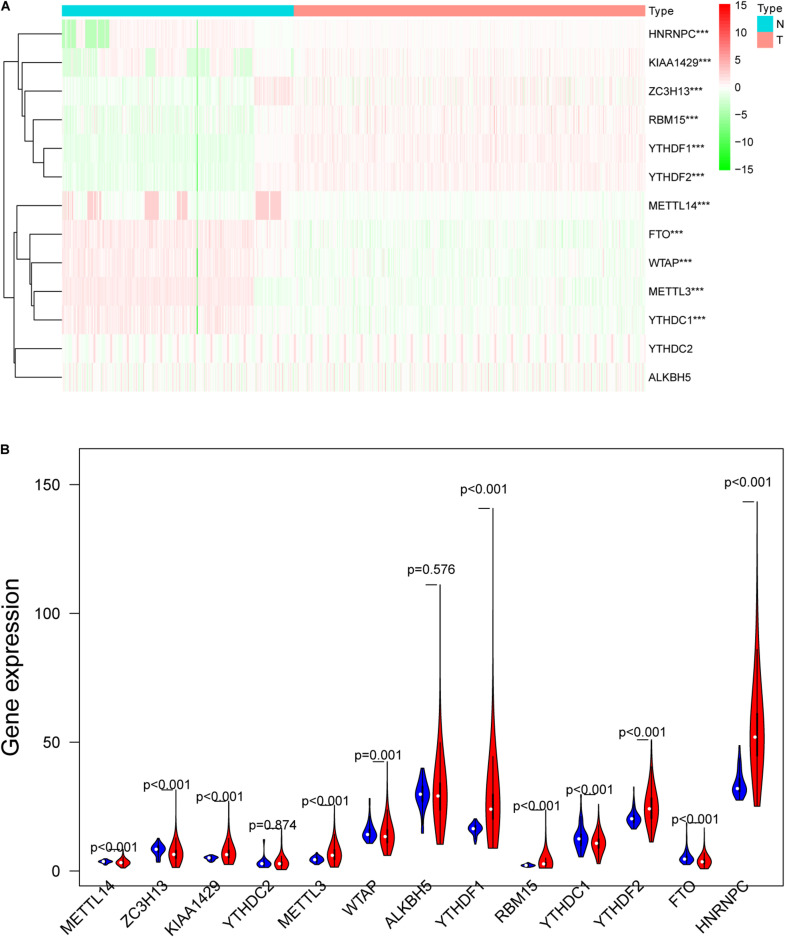
The differentially expressed m6A RNA methylation regulators between lung adenocarcinoma samples and normal control samples. **(A)** The heatmap was used to visualize the differential expression levels of m6A RNA methylation regulators between the cancer group and control group. **(B)** The violin plot was used to present the differential expression levels of m6A RNA methylation regulators between the cancer group and control group. N, normal samples; T, tumor samples; blue violins represent normal samples; red violins represent tumor sample; *P < 0.05, **P < 0.01, ***P < 0.001.

m6A RNA methylation regulators play an important role in tumorigenesis and development. In this study, expression levels of 13 m6A RNA methylation regulators in 535 lung adenocarcinoma tissues and 347 normal lung tissues from TCGA and GTEx database were investigated. The clinicopathological features of included lung adenocarcinoma cases were summarized in Table 1. Compared with the control group, expression levels of HNRNPC, YTHDF1, KIAA1429, RBM15, YTHDF2, and METTL3 in cancer group were significantly up-regulated (*P* < 0.05), while expression levels of FTO, ZC3H13, METTL14, YTHDC1, and WTAP in cancer group were significantly down-regulated (*P* < 0.05). The details were shown in Table 2. A heatmap and a violin plot were also used to visualize the differential expression levels of m6A RNA methylation regulators between the cancer group and control group (Figures 1A,B). No significant difference was found for expression levels of ALKBH5 and YTHDC2 between the two groups.

**TABLE 1:** The clinicopathological features of included lung adenocarcinoma cases in this study.

Clinicopathological features	Number
**Age**	
Mean	65.33 ± 8.21
**Gender, *n* (%)**	
Male	246(46.06%)
Female	289(53.94%)
**Pathological diagnosis**	
Lung adenocarcinoma	533(100%)
**TNM stage**	
Stage I	267(49.91%)
Stage II	143(26.73%)
Stage III	96(17.94%)
Stage IV	29(5.42%)

**TABLE 2:** The differential expression levels of 13 m6A RNA methylation regulators between lung adenocarcinoma samples and normal control samples.

m6A regulators	Expression levels		log_2_FC	*P*-value
	Adenocarcinoma	Control		
YTHDF1	26.289	15.063	0.803	1.881E-30
FTO	3.771	6.390	–0.761	3.569E-29
METTL14	3.318	3.458	–0.061	1.005E-27
YTHDF2	24.812	17.632	0.492	1.882E-20
ZC3H13	6.628	11.225	–0.761	5.698E-13
YTHDC1	11.143	19.587	–0.816	7.061E-13
RBM15	3.158	2.775	0.186	2.845E-08
METTL3	6.611	5.125	0.366	1.008E-05
HNRNPC	54.618	46.917	0.219	2.003E-05
KIAA1429	7.033	6.235	0.172	3.258E-04
WTAP	13.874	21.087	–0.606	1.058E-03
ALKBH5	29.995	35.956	–0.261	0.576
YTHDC2	3.013	2.774	0.119	0.874

### Interaction and Correlation Among the 13 m6A RNA Methylation Regulators

**FIGURE 2:**
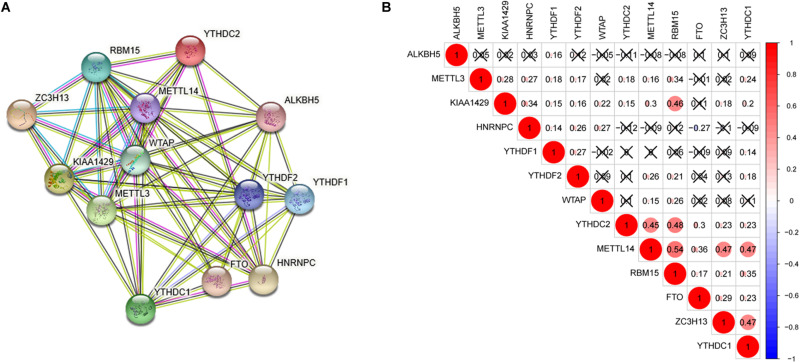
Interaction and correlation among the 13 m6A RNA methylation regulators. **(A)** The PPI network was constructed to show the interaction among m6A RNA methylation regulators. **(B)** The Pearson correlation analysis was used to evaluate the correlation among m6A RNA methylation regulators.

Interaction and correlation among the 13 m6A RNA methylation regulators were further analyzed to better understand the function of these regulators in pathogenesis of lung adenocarcinoma. As shown in Figure 2A, these genes interacted with each other to varying degrees and WTAP as well as METTL14 seemed to be the hub genes of the interaction network. Figure 2B presented that most of these regulators were positively related to each other weakly to moderately, and the strongest correlation was found between RBM15 and METTL14. In addition, a negative association was found between HNRNPC and FTO, indicating that when HNRNPC expression was up-regulated, FTO expression was likely to be down-regulated.

### Consensus Clustering of m6A RNA Methylation Regulators Identified 2 Subgroups of Lung Adenocarcinoma

**FIGURE 3:**
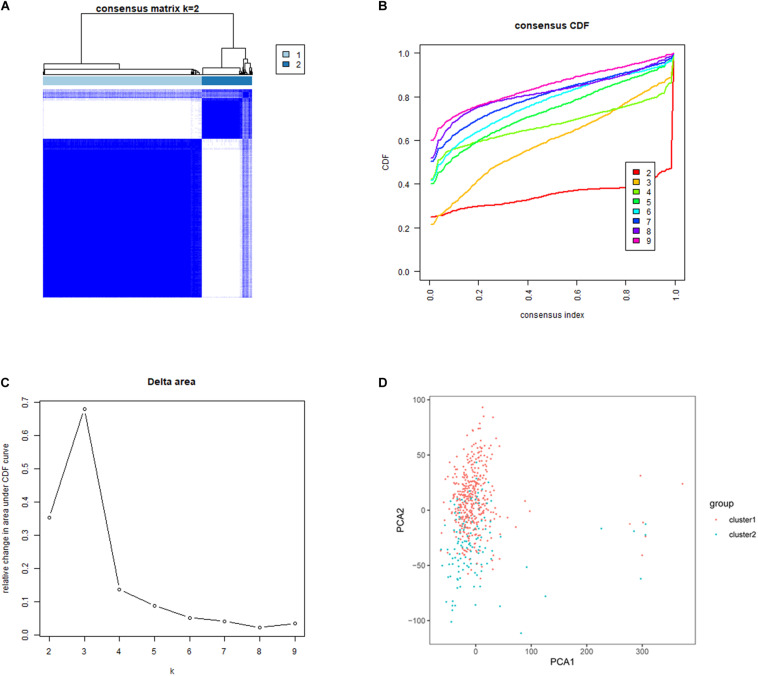
Identification of consensus clusters based on the expression similarity of m6A RNA methylation regulators. **(A)** Consensus clustering matrix for *k* = 2. **(B)** Consensus clustering cumulative distribution function (CDF) for *k* = 2–9. **(C)** Relative change in area under CDF curve for *k* = 2–9. **(D)** Principal component analysis (PCA) was used to verify the two distinct subgroups divided by consensus clustering of m6A RNA methylation regulators. Lung adenocarcinoma in cluster 1 subgroup was marked with red, while that in cluster 2 subgroup was marked with blue.

Five hundred and thirty-five lung adenocarcinoma tissues were grouped based on the expression similarity of m6A RNA methylation regulators. Figures 3B,C showed that 4 subgroups (*k* = 4) seemed to be a good choice with the smaller CDF value in TCGA database. However, there was a significant correlation among each group and 2 out of the 4 groups had a particularly small sample size. Therefore, we divided these cancer tissues into 2 subgroups, namely cluster 1 and cluster 2 (Figure 3A). Next, PCA was used to verify if the categories were appropriate. The result presented that both cluster 1 and cluster 2 gathered together, respectively (Figure 3D), indicating that the division of 2 subgroups was the better selection.

### Subgroups Identified by Consensus Clustering Were Closely Related to the Clinicopathological Features, Clinical Outcomes and Malignancy of Lung Adenocarcinoma

**FIGURE 4:**
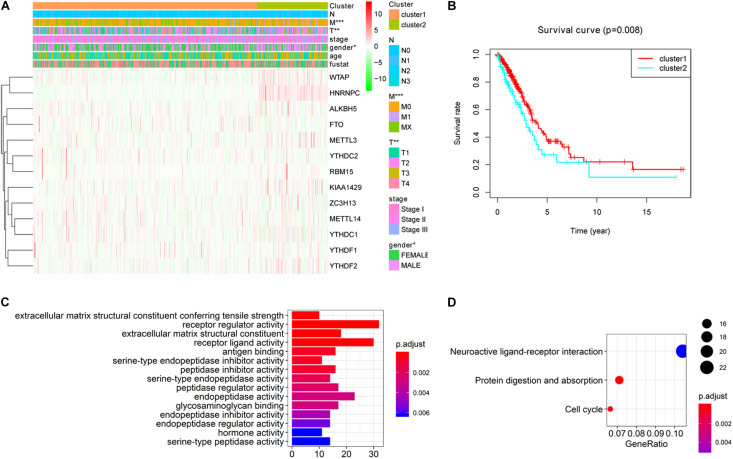
Differential clinicopathological features, clinical outcomes and malignancy of lung adenocarcinoma between cluster 1 and cluster 2. **(A)** Cluster 2 was found significantly correlated with male patients, higher T stage and higher M stage compared to cluster 1. **(B)** Significantly shorter overall survival (OS) was observed in cluster 2 than in cluster 1. **(C)** Gene ontology (GO) enrichment analysis of genes that were significantly dysregulated in cluster 2. **(D)** Kyoto Encyclopedia of Genes and Genomes (KEGG) pathway enrichment analysis of genes that were significantly dysregulated in cluster 2. *P < 0.05, **P < 0.01, ***P < 0.001.

To better understand the characteristic of subgroups identified by consensus clustering in lung adenocarcinoma, the clinicopathological features, clinical outcomes and malignancy of lung adenocarcinoma were compared between cluster 1 and cluster 2. Firstly, compared to cluster 1, the cluster 2 was found significantly correlated with male patients (*P* < 0.05), higher T stage (*P* < 0.01) and higher M stage (*P* < 0.05). The details were shown in Table 3 and Figure 4A. In addition, Figure 4B indicated that a significantly shorter OS was observed in cluster 2 than in cluster 1 (*P* < 0.01). Finally, genes that were significantly up-regulated (fold change > 8 and *P*-value < 0.01) or down-regulated (fold change > 8 and *P*-value < 0.01) in cluster 2 were identified and then annotated using GO and KEGG pathway analysis. GO analysis showed that these dysregulated genes in cluster 2 were enriched in many malignancy-related biological processes, such as extracellular matrix structural constituent conferring tensile strength, receptor regulator activity, receptor ligand activity, and so on (Figure 4C). KEGG analysis found that these dysregulated genes in cluster 2 were mainly involved in protein digestion and absorption, cell cycle and neuroactive ligand-receptor interaction signaling pathways (Figure 4D). These results indicated that subgroups identified by consensus clustering were closely related to the clinicopathological features, clinical outcomes and malignancy of lung adenocarcinoma.

**TABLE 3:** Different clinicopathological features between cluster 1 and cluster 2.

Clinicopathological features	Cluster 1 (*n*)	Cluster 2 (*n*)	*P*-value
**Gender**			0.013
Male	160	65	
Female	206	49	
**T stage**			0.003
T1	136	27	
T2	185	68	
T3	36	9	
T4	9	10	
**M stage**			0.024
M0	240	81	
M1	9	13	
Mx	117	20	

### Prognostic Value of m6A RNA Methylation Regulators and Identification of Prognostic Signature

**FIGURE 5:**
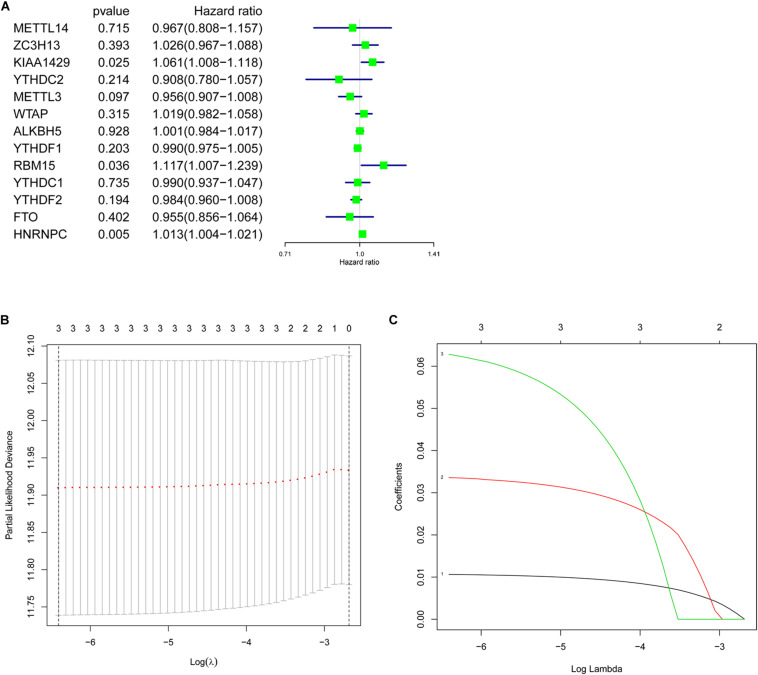
Prognostic value of m6A RNA methylation regulators and identification of prognostic signature. **(A)** Univariate Cox regression analysis indicated that KIAA1429, RBM15, and HNRNPC were risky genes for lung adenocarcinoma with the hazard ratio (HR) > 1. **(B,C)** The coefficients of genes selected to build prognostic signature were obtained using least absolute shrinkage and selection operator (LASSO) algorithm.

To better understand the prognostic value of m6A RNA methylation regulators in lung adenocarcinoma, the relationship of their expression levels and OS in TCGA database was identified using a univariate Cox regression analysis. The results presented that KIAA1429 (HR = 1.061, 95% CI = 1.008–1.118, *P* = 0.025), RBM15 (HR = 1.117, 95% CI = 1.007–1.239, *P* = 0.036), and HNRNPC (HR = 1.013, 95% CI = 1.004–1.021, *P* = 0.005) were significantly correlated with OS and they all were risky genes with the HR > 1 (Figure 5A). Next, these 3 genes were selected to build the prognostic signature (Figures 5B,C) and the coefficients were obtained using LASSO algorithm (Table 4). The risk score for each lung adenocarcinoma patient would be calculated with the following formula: risk score = 0.0335 ^∗^ KIAA1429 + 0.0628 ^∗^ RBM15 + 0.0106 ^∗^ HNRNPC. Finally, the lung adenocarcinoma patients in TCGA database were divided into high-risk group and low-risk group based on the median risk score.

**TABLE 4:** Genes selected to build risk signature and the corresponding coefficients.

Genes	Coefficients
HNRNPC	0.0106
KIAA1429	0.0335
RBM15	0.0628

### Prognostic Signature-Based Risk Score Was Not Only Strongly Associated With Clinical Outcomes and Clinicopathological Features, but Also an Independent Prognostic Factor in Lung Adenocarcinoma

**FIGURE 6:**
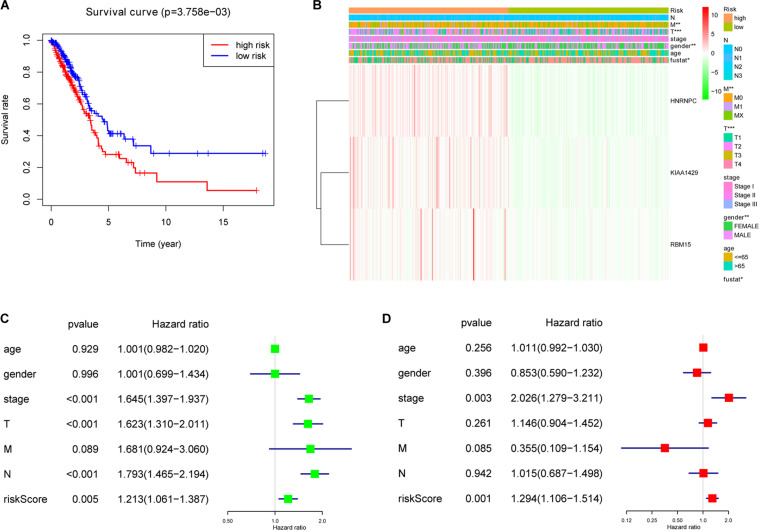
The association between risk score and clinical outcomes, clinicopathological features, as well as prognostic value of lung adenocarcinoma. **(A)** Lung adenocarcinoma patients in high-risk group had a significantly shorter overall survival (OS) than that in low- risk group. **(B)** Significant differences in terms of gender, T stage, M stage and survival state were observed between high-risk group and low-risk group. **(C)** Univariate Cox regression analysis presented that the whole stage, T stage, N stage, and risk score were significantly correlated with OS. **(D)** Multivariate Cox regression analysis further showed that the whole stage and risk score were the independent prognostic indicator for lung adenocarcinoma. *P < 0.05, **P < 0.01, ***P < 0.001.

To investigate prognostic value of the 3-gene risk signature in lung adenocarcinoma, the survival analysis was performed between the high-risk group and low-risk group. As shown in Figure 6A, the lung adenocarcinoma patients in high-risk group had a significantly shorter OS than that in low- risk group (*P* = 3.758e-03). Next, the relationship between risk scores and clinicopathological features was further evaluated. Figure 6B indicated that the lung adenocarcinoma patients in high-risk group generally contained a higher proportion of KIAA1429, RBM15, and HNRNPC than that in low-risk group. In addition, significant differences in terms of gender (*P* < 0.01), T stage (*P* < 0.001), M stage (*P* < 0.01), and survival state (*P* < 0.05) were also observed between high-risk group and low-risk group. However, no significant difference was found for age, N stage and the whole stage between high-risk group and low-risk group. The details were shown in Table 5 and Figure 6B. Then univariate and multivariate Cox regression analysis were performed to determine whether the prognostic signature-based risk score was an independent prognostic indicator for lung adenocarcinoma in TCGA database. The univariate analysis presented that the whole stage (HR = 1.645, 95% CI = 1.397–1.937, *P* < 0.001), T stage (HR = 1.623, 95% CI = 1.310–2.011, *P* < 0.001), N stage (HR = 1.793, 95% CI = 1.465–2.194, *P* < 0.001), and risk score (HR = 1.213, 95% CI = 1.061–1.387, *P* = 0.005) were significantly correlated with the OS (Figure 6C). While when these factors were added into the multivariate Cox regression model, only the whole stage (HR = 2.026, 95% CI = 1.279–3.211, *P* = 0.003) and risk score (HR = 1.294, 95% CI = 1.106–1.514, *P* = 0.001) were identified as the independent prognostic indicator for lung adenocarcinoma (Figure 6D). These results indicated that prognostic signature-based risk score was not only strongly associated with clinical outcomes and clinicopathological features, but also an independent prognostic factor in lung adenocarcinoma.

**TABLE 5:** Different clinicopathological features between the high-risk group and low-risk group.

Clinicopathological features	High-risk group (*n*)	Low-risk group (*n*)	*P*-value
**Gender**			0.001
Male	93	66	
Female	147	174	
**T stage**			< 0.001
T1	61	102	
T2	140	112	
T3	25	20	
T4	14	6	
**M stage**			0.009
M0	165	156	
M1	17	4	
Mx	58	80	
**Survival state**			0.011
Alive	110	145	
Dead	130	95	

## Discussion

Lung adenocarcinoma is one of the most aggressive and rapidly fatal tumors which needs better clinical management (Denisenko et al., 2018). Considerable effort should be made to prevent disease progression due to eventual acquired resistance of almost all targeted therapies (Ke et al., 2018; Wu and Shih, 2018; Yoneshima et al., 2018). m6A RNA methylation, which is shown essential for better prediction of malignant behavior and clinical prognosis of many cancers, has drawn attention in the exploration of earlier diagnosis and more effective treatment of lung adenocarcinoma (Taketo et al., 2018; Hu et al., 2019; Zou et al., 2019). In this study, most of the 13 m6A RNA methylation regulators were found abnormally expressed in 535 lung adenocarcinoma tissues from TCGA database. The relationship between these regulators and clinicopathological features was also analyzed and eventually a 3-gene risk signature was built. These results indicated that the m6A RNA methylation regulator played an important role in the pathogenesis of lung adenocarcinoma and could serve as a credible prognostic factor for lung adenocarcinoma.

Previous studies have shown that m6A RNA methylation regulators are involved in the pathogenesis of many types of tumors. For instance, FTO, a key m6A demethylase, is significantly overexpressed in human cervical cancer, breast cancer as well as acute myeloid leukemia and serves as an oncogenic regulator for the proliferation and migration of corresponding tumor cells (Li et al., 2017; Niu et al., 2019; Zou et al., 2019). m6A methyltransferase METTL3 is up-regulated in prostate cancer and bladder cancer cell lines, which promotes cell proliferation and survival, while silencing of METTL3 in these cell lines results in decreased cell proliferation and survival, suggesting that METTL3 contributes to the pathogenesis of prostate cancer and bladder cancer (Cai et al., 2019; Han J. et al., 2019). In addition, Choe et al. (2018) have claimed that METTL3 promotes translation of a large subset of oncogenic mRNAs via interacting directly with eukaryotic translation initiation factor 3 subunit h (eIF3h) (Choe et al., 2018). eIF3h is a subunit of eIF3 and is involved in the process of protein synthesis initiation (Marchione et al., 2013). Elevated eIF3h has been confirmed in 18% of breast cancer, 30% of prostate cancer (Nupponen et al., 1999) and 50% of hepatocellular carcinoma (Zhu et al., 2016). In lung adenocarcinoma, eIF3h protein was found up-regulated compared with normal lung tissues and an unfavorable factor promoting the cancer pathogenesis (Hu et al., 2020). Interestingly, Deng et al. have found that overexpression of METTL3 inhibits the proliferation, migration and invasion of colorectal cancer cells, while knockdown of METTL3 significantly reverses the effect on proliferation, migration and invasion, indicating that METTL3 plays a tumor-suppressive role in colorectal cancer (Deng et al., 2019). Our present analysis showed that among the 13 m6A RNA methylation regulators, 11 regulators were up-regulated or down-regulated in lung adenocarcinoma tissues, indicating that these genes might play key roles in the formation and development of lung adenocarcinoma. Further studies *in vivo* and *in vitro* are urgently needed to explore the underlying molecular mechanism.

In the present study, lung adenocarcinoma tissues were divided into two subgroups based on the expression similarity of m6A RNA methylation regulators, which were found associated with the pathological features of lung adenocarcinoma. Subsequently, the OS and tumor grades were shown significantly different between the two subgroups, indicating that the expression levels of m6A RNA methylation regulators were closely related to the prognosis of lung adenocarcinoma. In addition, these regulators were also demonstrated correlated with various biological processes and signaling pathways involved in the pathogenesis of lung adenocarcinoma. These biological processes included extracellular matrix structural constituent conferring tensile strength, receptor regulator activity as well as receptor ligand activity, and these signaling pathways mainly included protein digestion and absorption signaling pathway, cycle signaling pathway as well as cell neuroactive ligand-receptor interaction signaling pathway. Our above results are consistent with previous findings. Multiple m6A RNA methylation regulators have been identified involved in several malignancy-related biological processes such as tumor cell proliferation and migration, tumor immunity, RNA metabolism, DNA damage response as well as autophagy (Cui et al., 2017; Xiang et al., 2017; Dai et al., 2018; Jin et al., 2018; Deng et al., 2019; Han D. et al., 2019) and affecting many signaling pathways including p38/ERK pathway, β-catenin pathway as well as MYC/CEBPA pathway (Su et al., 2018; Zhou et al., 2018; Deng et al., 2019). Especially, it has been demonstrated that several m6A methyltransferase, such as METTL3 and YTHDF1, facilitate lung adenocarcinoma progression by activating cell migration and invasion through m6A methylation (Lin et al., 2016; Shi et al., 2019). While FTO and ALKBH5 affect the proliferation and invasion of lung adenocarcinoma cells via regulating multiple target genes and signaling pathways which are related to malignant tumors (Chao et al., 2020; Ding et al., 2020). The above studies have confirmed our present results to some extent that m6A RNA methylation regulators participate in the malignant progression of lung adenocarcinoma. Experiments *in vivo* and *in vitro* will be carried out in subsequent studies to prove our present findings.

Our study showed that the prognostic signature-based risk score, which was positively associated with the expression levels of KIAA1429, RBM15, and HNRNPC, was an independent prognostic factor for each lung adenocarcinoma patient, indicating that KIAA1429, RBM15, and HNRNPC all might function as oncogenes in lung adenocarcinoma. Current studies have demonstrated that high expression of KIAA1429 facilitates migration and invasion of hepatocellular carcinoma and is associated with poor prognosis among hepatocellular carcinoma patients (Cheng et al., 2019; Lan et al., 2019). Little is known about the role of RBM15 in tumorigenesis. Su et al. have found that RBM15 might contribute to malignant progression and have clinical prognostic impact in gastric cancer (Su et al., 2019). Similarly, it has been revealed that HNRNPC is abundantly expressed in colon adenocarcinoma and might play a vital role in the progression of colon adenocarcinoma (Liu T. et al., 2019). In addition, silencing of HNRNPC reduces cell proliferation and inhibits migratory and invasive activities of glioblastoma (Park et al., 2012), indicating that HNRNPC serves as a promoter in multiple tumors. Further studies *in vivo* and *in vitro* are required to explore the underlying molecular mechanism.

## Conclusion

Multiple m6A RNA methylation regulators were abnormally expressed and associated with the pathological features in lung adenocarcinoma. In addition, the prognostic signature-based risk score built according to the expression levels of KIAA1429, RBM15, and HNRNPC, was not only strongly associated with clinical outcomes and clinicopathological features, but also an independent prognostic factor in lung adenocarcinoma. Further studies *in vivo* and *in vitro* are required to identify these findings and to explore the underlying molecular mechanism.

## Data Availability Statement

The datasets generated in this study can be found in online repositories. The names of the repository/repositories and accession number(s) can be found in the article/supplementary material.

## Ethics Statement

Ethical review and approval was not required for the study on human participants in accordance with the local legislation and institutional requirements. Written informed consent for participation was not required for this study in accordance with the national legislation and the institutional requirements.

## Author Contributions

FL and YW designed the study. HW and HH analyzed and interpreted the data. LZ and DW organized the results. FL wrote the manuscript. All authors critically reviewed the manuscript and approved the final version.

## Conflict of Interest

The authors declare that the research was conducted in the absence of any commercial or financial relationships that could be construed as a potential conflict of interest.

## Abbreviations

ALK: anaplastic lymphoma kinase
CDF: cumulative distribution function
EGFR: epidermal growth factor receptor
GO: gene ontology
HR: hazard ratio
KEGG: Kyoto Encyclopedia of Genes and Genomes
LASSO: least absolute shrinkage and selection operator
m6A: methylation of N6 adenosine
OS: overall survival
PCA: principal component analysis
TCGA: The Cancer Genome Atlas.
